# AhR agonist tapinarof ameliorates lupus autoimmunity by suppressing Tfh cell differentiation via regulation of the JAK2‐STAT3 signaling pathway

**DOI:** 10.1002/iid3.903

**Published:** 2023-06-14

**Authors:** Ying Zhang, Yanyan Pan, Peiyi Zhang, Fang Wang, Ying Han, Kailin Li, Wen Jiang, Jue Wang, Yun Luan, Qian Xin

**Affiliations:** ^1^ Department of Respiratory Medicine The Second Hospital of Shandong University Jinan Shandong China; ^2^ Department of Pediatrics Qilu Children's Hospital of Shandong University Jinan Shandong China; ^3^ Department of Rheumatology and Immunology Jinan Central Hospital Jinan Shandong China; ^4^ Animal Laboratory Center, Institute of Medical Science The Second Hospital of Shandong University Jinan Shandong China; ^5^ Central Laboratory, Institute of Medical Science The Second Hospital of Shandong University Jinan Shandong China

**Keywords:** AhR, SLE, T follicular helper cell

## Abstract

**Background:**

The aryl hydrocarbon receptor (AhR) is a critical regulator of the pathogenesis of autoimmune disorders. We aimed to investigate the therapeutic effect of the AhR agonist tapinarof during the development of systemic lupus erythematosus (SLE).

**Methods:**

MRL/lpr mice were intraperitoneally injected with 1 or 5 mg/kg tapinarof for 6 weeks. Kidney histopathology was evaluated using hematoxylin and eosin (H&E) and Periodic‐Acid‐Schiff (PAS) staining. Immunofluorescence microscopy was performed to detect immune complex renal depositions. Flow cytometry (FCM) analysis was carried out to determine the proportions of T and B cell subsets. Realtime qPCR was used to quantify the expression of Tfh cell‐associated genes. We conducted an in vitro polarization experiment to observe the effect of tapinarof on Tfh differentiation. Western blotting was used to detect the expression of target proteins.

**Results:**

We found that tapinarof treatment ameliorated lupus phenotypes, including splenomegaly, lymph node enlargement, kidney damages, immune complex deposition, and excessive secretion of antibodies. Additionally, we showed that Treg subpopulation frequencies significantly increased in MRL/lpr mice treated with tapinarof, while the proportion of Th1/Th2 cells was reduced after tapinarof administration. Moreover, tapinarof suppressed Tfh cell differentiation and germinal center (GC) reaction in vivo. The inhibitory effect of tapinarof on Tfh cells was further verified in the in vitro Tfh cell polarization experiment. Realtime qPCR revealed that tapinarof repressed the expression of Tfh signature genes. Mechanistically, tapinarof significantly inhibited the phosphorylation levels of JAK2 and STAT3. The capacity for Tfh differentiation was partially rescued by the STAT3 activator Colivelin TFA. Furthermore, our in vitro Tfh polarization experiments indicated that tapinarof suppressed Tfh cell development in SLE.

**Conclusions:**

Our data demonstrated that tapinarof modulated the JAK2‐STAT3 pathway to suppress Tfh cell differentiation for the treatment of lupus symptoms in MRL/lpr mice.

## Graphical abstract

Tapinarof ameliorates lupus development of MRL/lpr mice. Tapinarof inhibits the differentiation of Tfh cells. Tapinarof modulates JAK2‐STAT3 pathway to suppress Tfh cell differentiation.

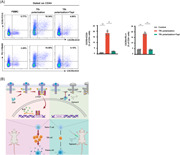

## 1 INTRODUCTION

Systemic lupus erythematosus (SLE) is a typical autoimmune disease characterized by excessive activation of lymphocytes and the generation of pathogenic autoantibodies, which contribute to immune complex deposition in multiple organs and lead to chronic inflammation and organ damage, such as renal failure, skin injury, and central nervous system (CNS) vasculitis.^1^ Extensive research efforts have been made that involve dysfunctions of numerous immune cells, such as Treg and Th17 cells, to investigate the pathogenesis of SLE.^2^ T follicular helper (Tfh) cells are a special population of helper T cells essential for the biological progression of plasma cell development, formation of germinal centers (GCs) as well as antibody production.^3^ Tfh cells express high levels of C‐X‐C chemokine receptor type 5 (CXCR5), programmed death‐1 (PD‐1), and inducible costimulatory molecule (ICOS). B‐cell lymphoma 6 (BCL6) is considered a key transcription factor for Tfh cell differentiation.^4^ It has been identified that Tfh cells are involved in the progression of autoimmune diseases and infection immunization.^5^

The aryl hydrocarbon receptor (AhR) is a ligand‐dependent nuclear transcription factor that belongs to the basic Helix–Loop–Helix/Per–Arnt–Sim (bHLH/PAS) family of toxin sensors.^6^ AhR signaling participates in processes related to pathophysiological changes in the human circadian rhythm, endocrine regulation, and tumorigenesis.^7^, ^8^, ^9^, ^10^ Recently, increasing evidence has shown that AhR serves as a critical regulator in the differentiation of immune cells and in the regulation of immune responses and secretion of cytokines.^11^, ^12^ AhR knockout mice manifest various phenotypic abnormalities, including a peripheral immune system disorder with decreasing total numbers of T and B cells in the spleen.^13^ D2,3,7,8‐tetrachlorodibenzo‐p‐dioxin (TCDD) and 6‐formylindolo[3,2‐b] carbazole (FICZ) are the most studied exogenous ligands of AhR.^14^ In vivo treatment with TCDD could ameliorate disease progression in several murine models of autoimmune diseases, such as uveoretinitis, colitis, and multiple sclerosis.^15^, ^16^, ^17^ Thus, AhR is considered to play an important role in the inflammation response and the occurrence and development of autoimmune disorders. However, the mechanisms by which AhR affects immune responses remain controversial, probably owing to differences in the cell‐specific functions of AhR and the different activating ligands.^18^

In recent years, the role of AhR and its ligands in the pathogenesis of SLE has received increasing attention. The expression level of AhR in mononuclear cells in the peripheral blood was substantially higher in patients with SLE than in healthy controls.^19^ The pharmacological manipulation of AhR activity has also been identified as an effective intervention for delaying the progression of spontaneous lupus‐like symptoms in mouse models.^20^ Consequently, screening and selecting effective therapeutic drugs that target AhR becomes essential for the treatment of SLE. In the current study, we investigated the therapeutic effects of AhR agonist tapinarof by administering its soluble form in the MRL/lpr lupus mouse model. Our results show that tapinarof‐treated lupus mice manifest moderate splenomegaly and lymph node enlargement, reduced kidney damage, and mild renal immune complex deposition, in addition to relative low concentrations of serum antibodies. We also demonstrate that tapinarof inhibits Tfh differentiation through the downregulation of JAK2 and STAT3 phosphorylation. Taken together, these results suggest that AhR is a new potential therapeutic target for SLE. Furthermore, these observations identify a previously unknown role for AhR in SLE therapy.

## 2 MATERIALS AND METHODS

### 2.1 Mice

Female MRL/lpr mice were purchased from Shanghai SLAC Laboratory Animal Co., Ltd. All mice were bred and maintained in specific pathogen‐free conditions at the Animal Laboratory Center of the Second Hospital of Shandong University. All experiments involving animals were approved by the Ethics Committee of the Second Hospital of Shandong University.

### 2.2 Tapinarof administration

The AhR agonist tapinarof (MCE) was dissolved in dimethyl sulfoxide (DMSO) and diluted in normal saline before the in vivo injections. MRL/lpr mice were intraperitoneally administered tapinarof at 1 or 5 mg/kg every other day from 14 to 20 weeks of age. The control group was treated with vehicle.

### 2.3 Histological staining and analysis

Fresh kidney tissues of mice were harvested after euthanasia and fixed overnight in formalin solution. The samples were then dehydrated and embedded in paraffin. Paraffin blocks were subsequently sectioned at 6 μm thickness on a microtome (Leica Biosystems). The sections were stained with hematoxylin and eosin (H&E) using an H&E Staining Kit (Beyotime) or with Periodic‐Acid‐Schiff (PAS) reagent using a PAS Staining Kit (Beyotime), following manufacturers' protocols.

### 2.4 Immunofluorescence microscopy

Mice kidneys were collected and frozen in Tissue‐Tek Optimal Cutting Temperature Compound on dry ice and were immediately stored at −80°C. Kidney cryosections (7 μm) were fixed with cold acetone for 10 min, and 0.3% Triton X‐100 in PBS for 20 min was used for permeabilization. The sections were stained with IgG‐AF565 and IgM‐AF488 antibodies (Thermo Fisher Scientific) and incubated in 37°C for 2 h. Nuclei were counterstained with DAPI (Beyotime). Fluorescent images were captured with a ZEISS LSM 800 confocal microscope (Carl Zeiss). Immunofluorescence images were analyzed using the Quantity One software to evaluate mean fluorescence intensity.

### 2.5 Flow cytometry (FCM) analysis

Single‐cell suspensions were prepared by passing spleens or lymph nodes through a 70‐µm cell strainer (BD). After centrifugation at 200*g* for 10 min, red blood cells were removed using red blood cell lysis buffer (Tiangen) for 10 min. The lysis buffer was neutralized by washing once with PBS. Splenocytes and lymph node cells were then labeled with monoclonal antibodies and analyzed on the Beckman flow cytometer (Beckman Coulter). Intracellular cytokine staining was performed using the Foxp3/Transcription Factor Staining Buffer and Intracellular Fixation & Permeabilization Buffer Sets (eBioscience) for the detection of Th1, Th2, and Th17 cells. The following flow‐cytometry antibodies were used in the present study: CD4‐FITC, CD25‐APC, FOXP3‐PE, IFN‐γ‐PC5.5, IL‐4‐PE, IL‐17a‐APC, CXCR5‐ECD, ICOS‐PC5.5, GL‐7‐APC, FAS‐PE, B220‐FITC, and CD138‐BV421. All fluorescent‐conjugated antibodies used in this study were purchased from Biolegend.

### 2.6 Detection of cytokines

We determined the concentrations of immunoglobulin subtypes in mouse plasma samples using the LEGENDplex assay (BioLegend). Plasma cytokine profiles were detected using the Mouse Th1/Th2/Th17 Cytometric Bead Array (CBA) Kit (BD). Both kits were applied following the manufacturers' protocols.

### 2.7 Enzyme‐linked immunosorbent assay (ELISA)

For plasma IL‐21 determination, ELISA plates (Thermo Fisher Scientific) were coated with anti‐mouse IL‐21 purified capture antibody and incubated overnight at 4°C. After three washes with wash buffer, the samples and standards were added to the plates and incubated at room temperature for 2 h. This was followed by a 30‐min incubation in detection antibody dilution at room temperature. After washing the wells three times, diluted avidin‐HRP solution was added, and incubation continued at room temperature for 30 min. Subsequently, TMB substrate solution was added to each well, and the reactions were allowed to proceed for 15 min at room temperature. The plate absorbance at 450 nm was determined within 1 h after addition of the stop solution.

### 2.8 Detection of blood urea nitrogen (BUN) levels

BUN levels were assessed using the Urea Nitrogen Colorimetric Detection Kit (Thermo Fisher Scientific) following manufacturers' instructions.

### 2.9 RNA isolation and quantitative real‐time PCR

Total RNA was collected from the mouse Tfh polarized cells using the TRIzol Reagent (Invitrogen) according to the manufacturer's instructions. Complementary DNA (cDNA) was synthesized using the PrimeScript RT Reagent kit (Takara). Quantitative real‐time PCR was performed on a QuantStudio™ 5 Real‐Time PCR System (Applied Biosystems) to detect the expression of several genes. Glyceraldehyde‐3‐phosphate dehydrogenase (GAPDH) gene expression was used as an internal standard to normalize target gene mRNA levels in quantitative real‐time PCR. The sequences of the real‐time primers are listed in Table 1. The thermal cycling conditions for the RT‐qPCR were designed as follows: 95°C for 1 min, followed by 40 cycles of 95°C for 5 s, 60°C for 10 s, and 72°C for 15 s. The $2^{-\Delta \Delta C_{t}}$method was used to calculate the relative changes of gene expression by employing the cycle threshold (*C*_t_) values. The results are presented as the mean ± standard deviation (SD).

**Table 1:** The primer sequences for RT‐qPCR.

Primer name	Forward primer	Reverse primer
Bcl6	CCGGCACGCTAGTGATGTT	TGTCTTATGGGCTCTAAACTGCT
Cxcr5	ATGAACTACCCACTAACCCTGG	TGTAGGGGAATCTCCGTGCT
Icos	ATGAAGCCGTACTTCTGCCAT	CGCATTTTTAACTGCTGGACAG
Il‐6	CCTTGGATAGAGCCCAGGAC	GGTCGTCTTGCTTTCCTTCTC
CD40l	GCTGAACTGTGAGGAGATGAG	CCACTGTAGAACGGATGCTG
Il‐7r	GCGGACGATCACTCCTTCTG	AGCCCCACATATTTGAAATTCCA
Maf	GGAGACCGACCGCATCATC	TCATCCAGTAGTAGTCTTCCAGG
Gapdh	AGGTCGGTGTGAACGGATTTG	TGTAGACCATGTAGTTGAGGTCA

### 2.10 In vitro Tfh cell differentiation

Regarding the protocol for the in vitro mouse Tfh polarization assay, 24‐well round‐bottom tissue culture plates were incubated with 4 μg/mL anti‐CD3 antibody (BioLegend) in PBS at 4°C overnight. Single‐cell suspensions from mouse spleens were prepared, and the red blood cells were lysed with red blood cell lysate (BD). 5 × 10^5^ isolated splenocytes were transferred per well onto the precoated 24‐well round‐bottom tissue culture plates and additionally activated with 1 μg/mL anti‐CD28 (BioLegend, USA), 10 μg/mL anti‐IFN‐γ (BioLegend), 10 μg/mL anti‐IL‐4 antibodies (BioLegend, USA), 20 μg/mL anti‐TGF β (BioLegend), and 10 ng/mL each IL‐6 and IL‐21 (PeproTech) cytokines for 3 days. The percentage of in vitro‐polarized Tfh cells was analyzed using flow cytometry. For the induction of human Tfh cells in vitro, 24‐well plates were precoated with 4 μg/mL anti‐CD3 antibody (OKT3) (BioLegend) at 4°C overnight. The cells were then incubated on the precoated plates in presence of 1 μg/mL anti‐CD28 antibody (BioLegend), 20 μg/mL recombinant human IL‐21 (PeproTech), 5 μg/mL recombinant human IL‐12 (PeproTech), 1 μg/mL recombinant human TGF‐β (PeproTech), and 20 μg/mL recombinant human IL‐6 (PeproTech) at 37°C under 5% CO_2_ conditions for 3 days. Plated cells were treated with 0.1 mΜ tapinarof or 50 µM Colivelin TFA as indicated.

### 2.11 Western blotting assay

Protein samples were extracted from mouse spleens using a radioimmunoprecipitation assay (RIPA) buffer supplemented with a protease and phosphatase inhibitor cocktail (Roche). Protein concentration was determined using the BCA method (Thermo Fisher Scientific). Protein extracts (20 μg) were loaded onto and separated in 12% SDS‐PAGE gels, and then electrotransferred to polyvinylidene difluoride (PVDF) membranes. The membranes were blocked with 5% skimmed milk (BD) and incubated with the specific primary antibodies P‐JAK2, P‐STAT3, or β‐actin (Cell Signaling) overnight at 4°C, followed by incubation with HRP‐conjugated secondary antibodies for 1 h at room temperature. Immunoreactivity was detected using the ECL prime reagent (Millipore) and chemiluminescence signals were recorded on a chemiluminescence gel imaging system (Tanon).

### 2.12 Statistical analysis

All statistical analyses were conducted using the GraphPad Prism version 8 software (GraphPad Software). For comparisons between multiple groups, the nonparametric Kruskal–Wallis test followed by the uncorrected Dunn's multiple comparison test was used. The data are expressed as means ± SD. A *p* < .05 was considered to indicate statistical significance. The schematic model was created using the online tool BioRender (https://biorender.com/).

## 3 RESULTS

### 3.1 Tapinarof inhibited autoimmune manifestation in MRL/lpr mice

**Figure 1:**
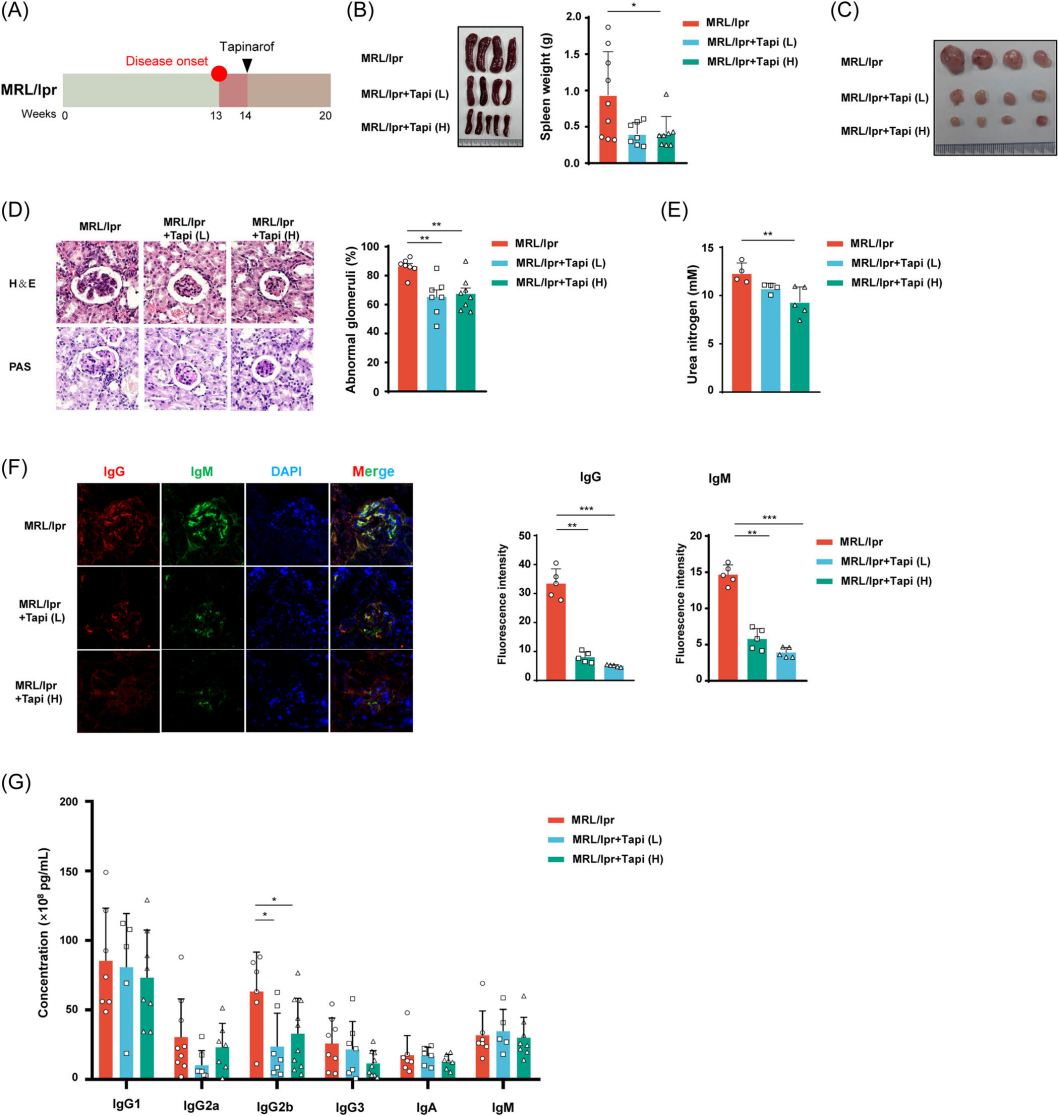
Tapinarof inhibits autoimmune aggravation in MRL/lpr mice. (A) Schematic diagram showing the experimental design of the present study. (B) The spleens of tapinarof‐treated and untreated mice were removed, and the weights were measured (*n* = 7–9). (C) Cervical lymph nodes were isolated from the mice (*n* = 7–9). (D) Representative H&E‐ and PAS‐ stained images from mouse kidney sections were collected at the end of the observation period. Scale bars: 50 μm (left). The numbers of abnormal glomeruli were recorded (right) (*n* = 7–8). (E) The plasma urea nitrogen levels were measured (*n* = 4–5). (F) IgG and IgM depositions in the kidney sections were assessed using immunofluorescence staining (left). The mean fluorescence intensity (MFI) was analyzed with ImageJ (right) (*n* = 5). (G) Antibody titers of immunoglobulin classes were detected using the LEGENDplex kit (*n* = 5–10). The data were obtained from two independent experiments. Error bars indicate SD. Each symbol in the graphs represents a separate mouse; Tapi (L), low concentration (1 mg/kg) of tapinarof; Tapi (H), high concentration (5 mg/kg) of tapinarof; *p < .05, **p < .01, and ***p < .001.

As one of the best‐studied animal models of human SLE, the MRL/lpr mouse strain spontaneously develops a severe autoimmune syndrome, characterized by splenomegaly, glomerulonephritis, lymphadenopathy as well as increased autoantibody production.^21^ To investigate whether the AhR agonist tapinarof could ameliorate SLE clinical features, 14‐week‐old MRL/lpr mice were intraperitoneally administered different concentrations of tapinarof or vehicle every other day for 6 weeks (Figure 1A). Compared with vehicle‐treated mice, treatment with a high dose (5 mg/kg) of tapinarof markedly reduced spleen size and spleen weight (Figure 1B). Furthermore, a reduction in the size of cervical lymph nodes in the tapinarof treatment group was recorded (Figure 1C). H&E and PAS staining of kidney sections revealed a lower proportion of abnormal glomeruli in the tapinarof‐treated mice than in the vehicle group (Figure 1D). This compound also decreased the level of BUN (Figure 1E). Immunofluorescence staining showed that IgG and IgM deposition was reduced in the glomeruli of kidneys both in low‐ and in high‐concentration tapinarof‐treated mice (Figure 1F). The above data demonstrate that tapinarof can improve renal functions during lupus progression. Consistent with these findings, the plasma titers of IgG2b decreased in the treatment group (Figure 1G). These results suggest that tapinarof can relieve lupus nephritis in MRL/lpr mice.

### 3.2 Tapinarof affected the Th cell population

**Figure 2:**
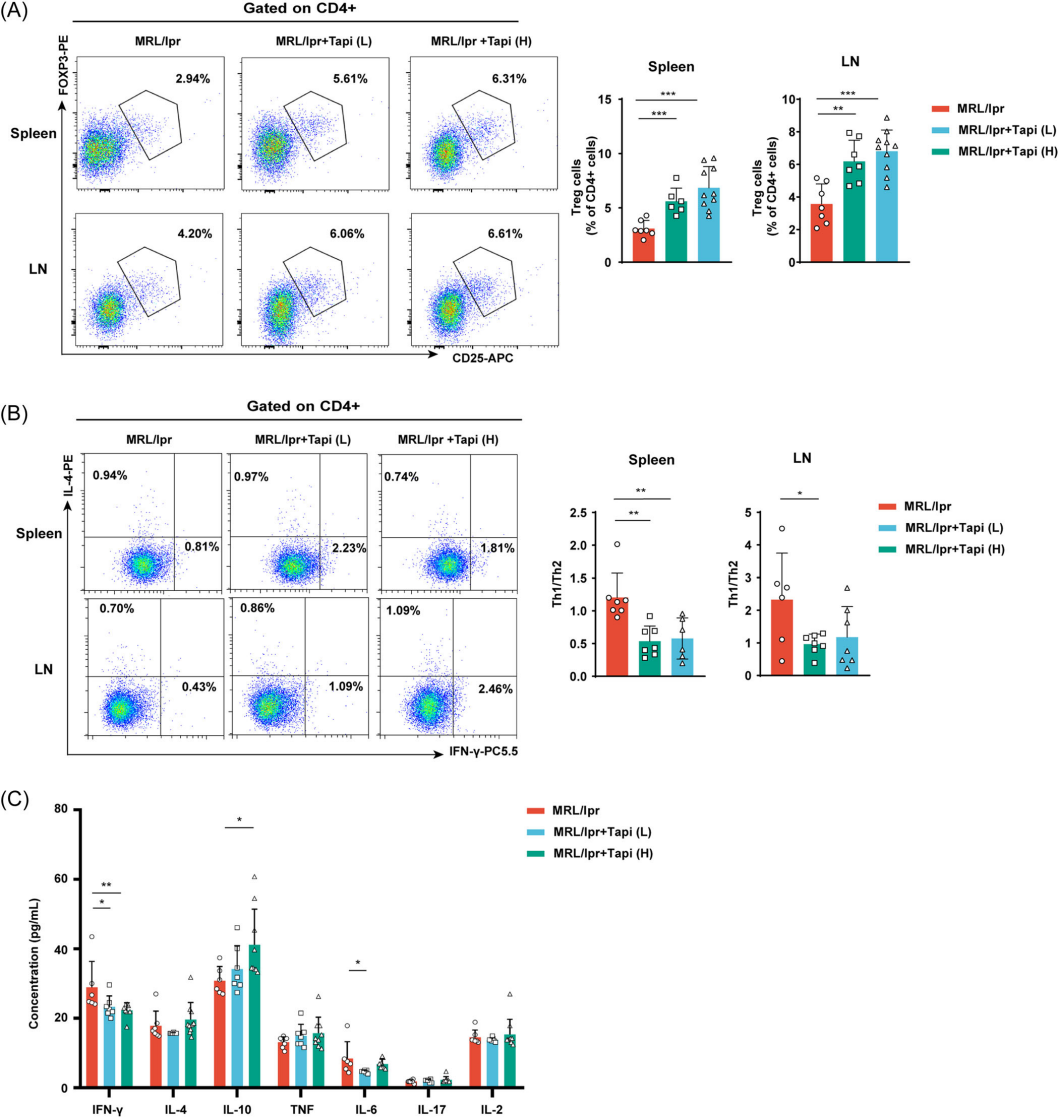
Effects of tapinarof treatment on SLE‐related immune cells. (A) Representative FACS pseudo‐color plots show Treg cell populations (CD4+CD25+FOXP3+) in spleens and lymph node cells. The results of the statistical analysis are shown on the right (*n* = 6–10). (B) Representative FACS pseudo‐color plots for Th1 (CD4+IFN‐γ+) and Th2 cells (CD4+IL‐4+) (left) and the ratio of Th1/Th2 cells (right) (*n* = 6–7). (C) The histogram shows the concentrations of Th1/Th2/Th17‐related cytokines in the plasma in each study group (*n* = 6–10). The data were obtained from two independent experiments. Error bars indicate SD; *p < .05, **p < .01, and ***p < .001. SLE, systemic lupus erythematosus.

In various autoimmune diseases, AhR activation regulates the differentiation of Th cells. Activation of AhR, for example, induced transcription factor FOXP3 and Treg cell development.^22^ In our study, we observed a higher frequency of Treg cells in the tapinarof‐treated group than in untreated MRL/lpr mice (Figure 2A). We detected a higher proportion of Th2 (CD4+IL‐4+) cells in the spleens of mice treated with 1 mg/kg tapinarof (Supporting Information: Figure 1B). In addition, higher frequencies of Th1 (CD4+IFN‐γ+) and Th2 cells were observed in the lymph nodes of mice treated with 1 mg/kg and 5 mg/kg, respectively (Supporting Information: Figure 1C). Further analysis of the flow cytometry data showed a significant decrease in the Th1/Th2 ratio in the spleens and lymph nodes in the high‐concentration tapinarof‐treated mice (Figure 2B). The CBA assay was used to assess the levels of Th1/Th2/Th17 cell‐related cytokines in the plasma. Tapinarof‐treated mice displayed higher levels of the anti‐inflammatory cytokine IL‐10 and lower levels of pro‐inflammatory cytokines, IFN‐γ and IL‐6 (Figure 2C). The representative manual gating schema used to identify CD4+ T cell subsets, including Treg and Th1 and Th2 cells, is shown in Supporting Information: Figure 1A. Although we did not exclude dead and non‐T cells that could affect the precision of our flow cytometry data, our findings do imply that tapinarof can control Treg cell differentiation and Th1/Th2 homeostasis, thereby restoring immune homeostasis.

### 3.3 Tapinarof decreased the Tfh cell population in MRL/lpr mice

**Figure 3:**
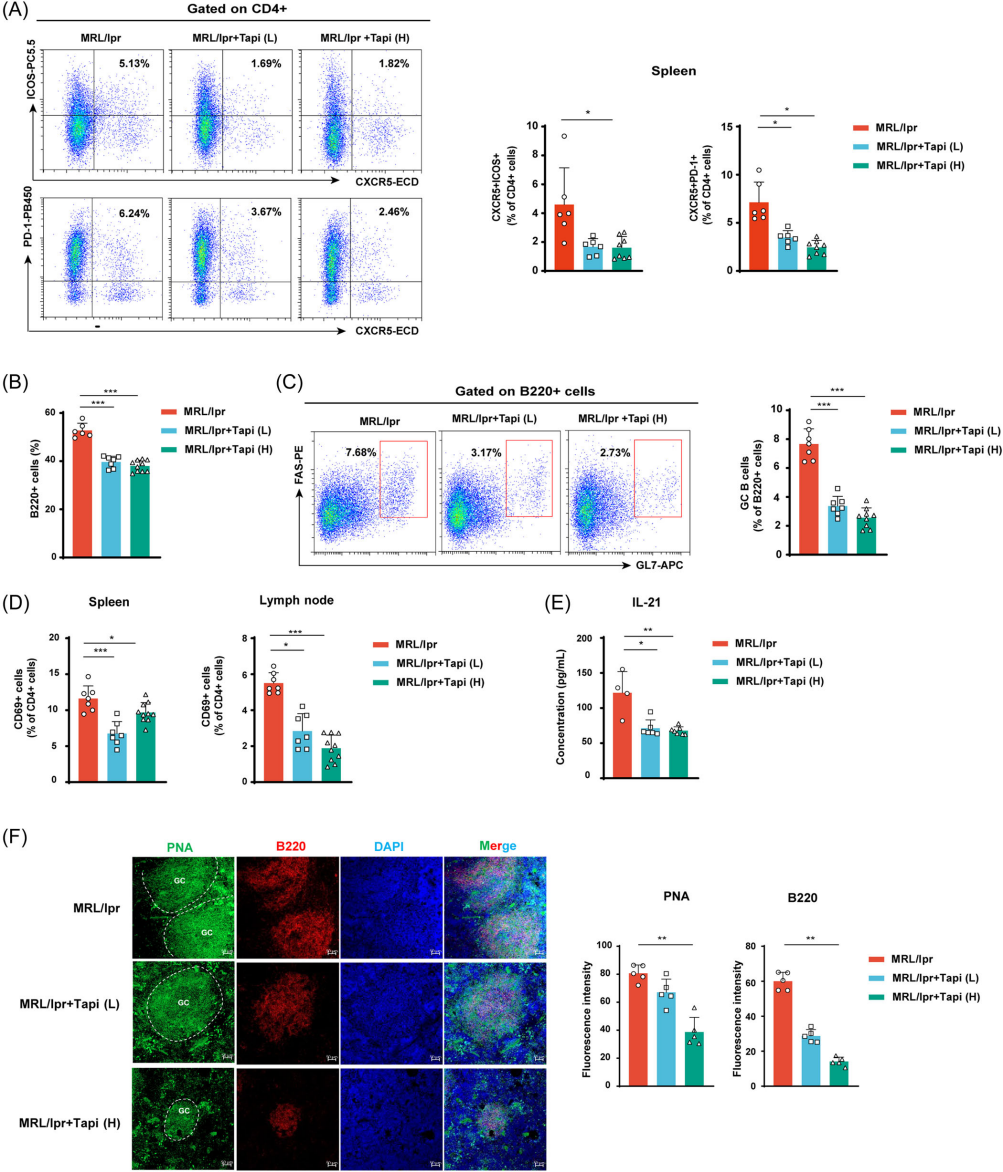
Tapinarof treatment reduces Tfh expansion and GC response in MRL/lpr mice. (A) Representative FACS pseudo‐color plots of the Tfh cell population (left). The results of the statistical analysis are shown on the right (*n* = 6–8). (B) Proportion of total B cells in the spleens of the three studied groups (*n* = 6–10). (C) Representative FACS pseudo‐color plots and the percentage of GC B cells in spleens (*n* = 7–9). (D) Proportion of CD4+CD69+ cells in the spleens and lymph nodes of the three studied groups (*n* = 7–10). (E) IL‐21 concentrations were detected in the plasma using an ELISA kit (*n* = 4–7). (F) Immunofluorescence of GCs from representative images of mouse spleen sections stained with PNA (Alexa Fluor 488 conjugate), B220 (Alexa Fluor 568 conjugate), and DAPI among the three different treatments. The results of the statistical analysis are shown on the right (*n* = 5). The data were obtained from two independent experiments. Error bars indicate SD; *p < .05, **p < .01, and ***p < .001.

Expansion of Tfh cell populations is associated with the progression of autoimmune diseases, including SLE. In the present study, we assessed the effect of tapinarof‐mediated AhR activation on regulating Tfh cell differentiation in vivo. Our results showed that tapinarof treatment clearly reduced the percentage of splenic Tfh cells (Figure 3A). Furthermore, we observed significant reductions in the frequencies of total B cells and GC B cells in the spleens of tapinarof‐administered mice (Figure 3B,C and Supporting Information: Figure 2C). Compared with the control, tapinarof‐treated mice exhibited a decreased proportion of activated T cells (CD4+CD69+) (Figure 3D and Supporting Information: Figure 2D). As IL‐21 is an important factor in GC formation and auto‐reactive plasmablast production, we assessed IL‐21 levels in the tapinarof‐treated and untreated MRL/lpr mice. ELISA results showed that the plasma IL‐21 concentration decreased significantly after tapinarof treatment (Figure 3E). We also found that high‐dose tapinarof treatment inhibited the differentiation of GC B cells, identified by the PNA+B220+ phenotype in splenic slices (Figure 3F). Supporting Information: Figure 2A,B shows an example of the flow cytometry gating strategy used to identify T and B cell subsets. While a viability dye and T‐cell specific markers should be used to verify our data in further studies, our results suggest that tapinarof effectively reduced the Tfh cell response in MRL/lpr mice.

### 3.4 Tapinarof inhibited the differentiation of Tfh‐like cells through JAK2‐STAT3 signaling

**Figure 4:**
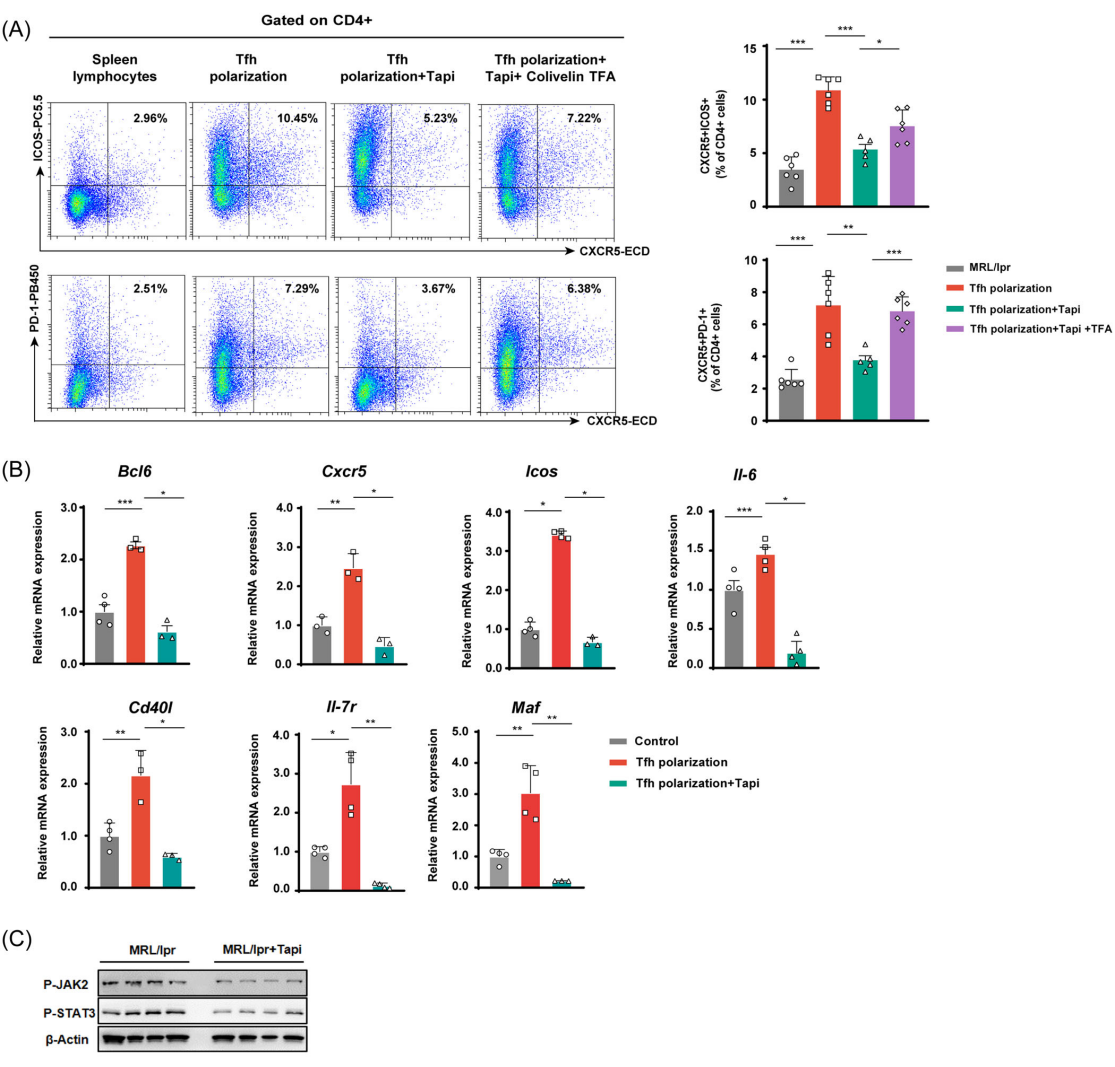
Tapinarof treatment affects Tfh expansion in vitro through JAK2‐STAT3 signaling. Spleen lymphocytes were isolated from MRL/lpr mice and cultured for 3 days under different treatment conditions. (A) Pseudo‐color plots in the left panel show the percentages of Tfh cell subsets in gated CD4+ T cells in each study group. The results of the statistical analysis are shown on the right (*n* = 5–6). (B) Real‐time qPCR analysis of *Bcl6*, *Cxcr5*, *Icos*, *Il‐6*, *CD40l*, *Il‐7r*, and *Maf* mRNA expression in each study group (*n* = 3–4). qPCR analysis was performed in triplicate in three separate runs for each gene expression. (C) P‐JAK2 and P‐STAT3 immunoblots in the spleens of tapinarof‐ treated and control mice. The data were obtained from two independent experiments. Error bars represent SD. Each symbol in the graphs represents an individual mouse; *p < .05, **p < .01, and ***p < .001.

Subsequently, we sought to investigate the role of tapinarof in regulating Tfh cell differentiation in vitro. Splenocytes were cultured in vitro under Tfh‐polarizing conditions for 3 days. We confirmed that Tfh cell differentiation was inhibited by tapinarof in vitro (Figure 4A).

To evaluate the effects of tapinarof on the expression patterns of Tfh cell signature genes, Tfh cell‐related genes were detected using realtime qPCR in the three groups. We found that tapinarof negatively regulated the expression of multiple Tfh‐associated genes, including *Bcl6*, *Cxcr5*, *Icos*, *Il‐6*, *Cd40l*, *Il‐7r*, and *Maf* (Figure 4B). Collectively, the in vitro experiments demonstrated that tapinarof exhibited an inhibitory role in Tfh cell differentiation.

JAK‐STAT signaling exerts important developmental effects on the differentiation of helper T cell subpopulations.^23^ Given that AhR could mediate STAT3 signaling, P‐JAK2 and P‐STAT3 expression levels were detected in the spleens of drug‐treated and untreated MRL/lpr mice. The results showed that the protein levels of both P‐JAK2 and P‐STAT3 were downregulated in the tapinarof administration group (Figure 4C). The STAT3 activator Colivelin TFA was used in the Tfh polarization experiment. The results showed that Colivelin TFA rescued the differentiation of tapinarof‐treated Tfh cells (Figure 4A), emphasizing the involvement of the JAK2‐STAT3 signaling pathway in tapinarof mediated effects. Nevertheless, further studies with, for example, more detailed phenotyping are warranted to confirm the findings of our research.

### 3.5 Effect of tapinarof on peripheral blood mononuclear cells (PBMCs) of patients with SLE

**Figure 5:**
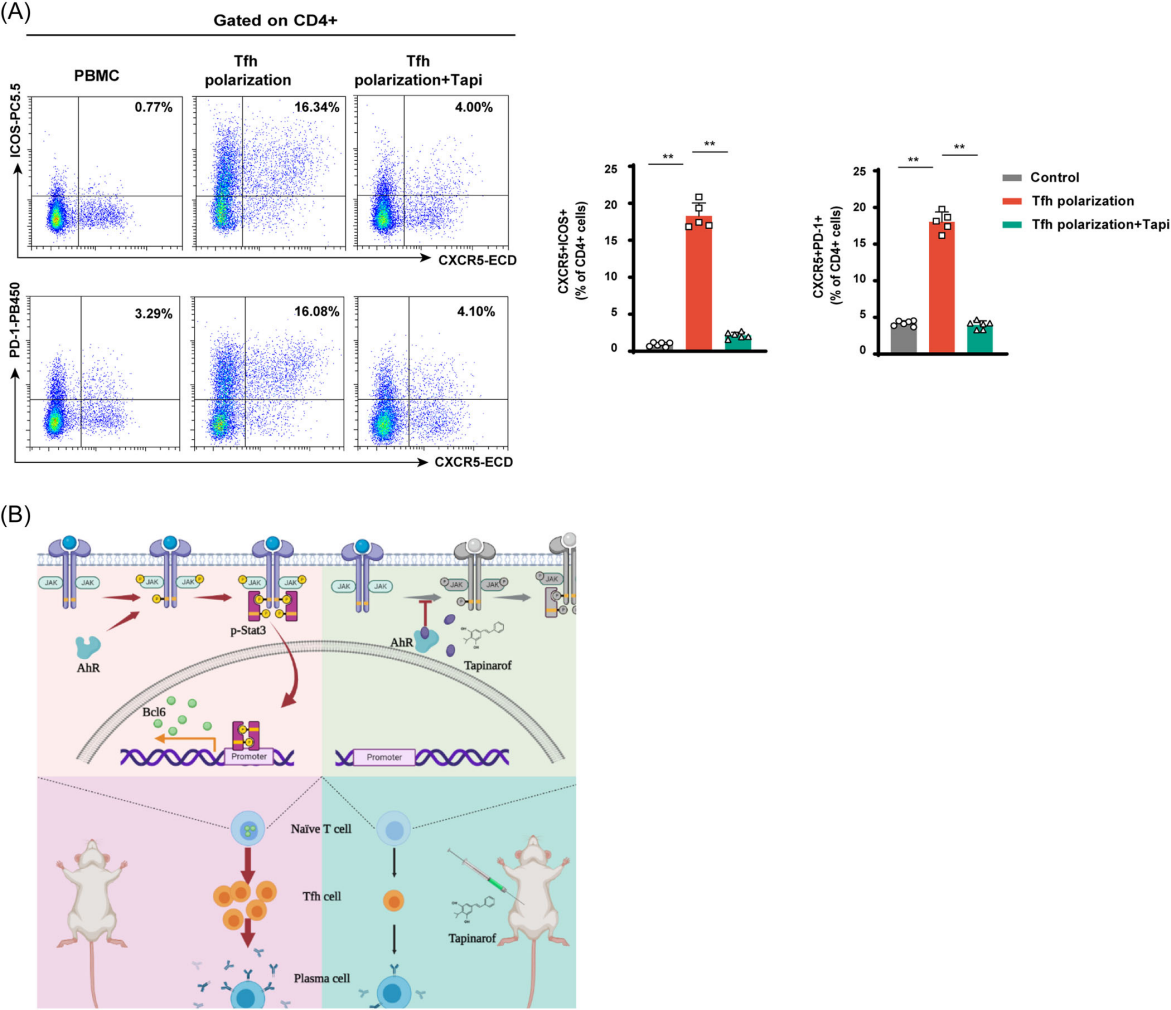
Tapinarof inhibits Tfh cell differentiation in patients with SLE. (A) PBMCs from patients with SLE were cultured in the different polarization conditions for 3 days and percentages of Tfh cells were investigated (*n* = 5–6). (B) Schematic model of the therapeutic effect of tapinarof on the lupus syndrome in MRL/lpr mice. The data were obtained from two independent experiments. Error bars represent SD. Each symbol in the graphs represents one sample; **p < .01. SLE, systemic lupus erythematosus.

To investigate the function of tapinarof in regulating human Tfh cell differentiation, PBMCs were isolated from patients with SLE. PBMCs were cultured under Tfh polarization conditions. The gating strategy for the human Tfh cells is illustrated in Supporting Information: Figure 3A. Flow cytometry analysis showed that the percentage of Tfh cells decreased after tapinarof treatment (Figure 5A). Our results revealed that tapinarof exerted an inhibitory effect on the differentiation of human Tfh cells.

## 4 DISCUSSION

In this study, tapinarof improved renal histopathology and reduced the production of autoantibodies and cytokines in MRL/lpr mice. Tapinarof also maintained the balance of the Th1/Th2 ratio and enhanced Treg cell development. Notably, the differentiation of Tfh cells was significantly inhibited by tapinarof administration in vivo and in vitro. These effects of tapinarof were mediated by the inhibition of JAK2‐STAT3 signaling. On the basis of these observations, we revealed that the AhR modulator tapinarof/AhR‐JAK2‐STAT3 signaling exerted an immunosuppressive effect on SLE by suppressing Tfh cell differentiation (Figure 5B). The results obtained from these experiments enhance our understanding on the role of AhR in the treatment of SLE by regulating Tfh cell development.

Our study suggests that tapinarof exerts anti‐inflammatory effects in an animal model of SLE. In a subsequent step, it would be worth exploring potential side effects of tapinarof treatment in the SLE mouse model. Here, we observed that treatment with tapinarof decreased splenic weight and exhibited protective effects on MRL/lpr mice. Additionally, we found that tapinarof alleviated kidney inflammation in SLE mice through kidney pathological evaluation and BUN level detection. Our preliminary results suggest that no adverse renal effects occurred during tapinarof treatment, at least in mice. However, evaluation of kidney function should be confirmed in further studies using other functional tests, such as the creatinine and glomerular filtration rate. Moreover, functional tests should be conducted to evaluate the levels of the liver enzymes GPT, ALP, and GOT. Additionally, the overall health status of the mice should be estimated using body weight curves.

The dysregulation and expansion of Tfh cells contribute to the pathogenesis of SLE. One of the important discoveries of the current study was that modulating AhR could alter Tfh cell differentiation and thus GC responses in the SLE mouse model. Our findings suggest that AhR may be a potential therapeutic target for the treatment of SLE. Other than autoimmune diseases, previous studies have demonstrated that AhR activation by KYNA, ITE, as well as TCDD remarkably decreased the frequency of Tfh cells in mice infected with the influenza virus. Furthermore, T cell‐specific AhR knockout (KO) virus‐infected mice did not manifest changes in the percentage and number of Tfh cells after AhR activation.^24^ Our results and previous reports indicate the important role of AhR in Tfh cell differentiation in pathological conditions. As a specific population characterized by CD4+CXCR5+PD‐1+FOXP3+, T follicular regulatory (Tfr) cells are considerably antagonistic to Tfh cells and could negatively regulate GC responses.^25^ It would be of interest to investigate Tfr cells as well as the balance between Tfh and Tfr cells in future studies.

AhR has also been found to be essential for the development of Treg cells. The functions of Treg cells have been well‐established in both patients with SLE and animal models. TCDD treatment contributes to the induction of the population and enhances the suppressive function of Treg cells in an AhR‐dependent manner in both mice and humans.^16^, ^26^ The endogenous AhR ligand ITE has also been demonstrated to suppress autoimmunity by inducing Treg cells.^27^ Quintana et al.^22^ found that ITE acts on dendritic cells (DCs) and T cells to promote the generation of functional FOXP3+ Treg cells, thereby inhibiting the progression of experimental autoimmune myelitis (EAE), which is a mouse model of multiple sclerosis (MS). Further studies are needed to investigate whether and how AhR ligands regulate Treg cells involved in SLE.

The AhR‐modulating agent tapinarof (GSK2894512) is a promising naturally derived drug molecule for the treatment of atopic dermatitis (AD) and plaque psoriasis.^28^, ^29^ Tapinarof is a direct binding partner and agonist ligand of AhR.^30^ Animal models and clinical experiments have confirmed the immunosuppressive effects of tapinarof on skin inflammation.^30^ Here, we first investigated the potential therapeutic value of tapinarof agents in the lupus mouse model. The current study examined whether treatment with low‐ and high‐dose tapinarof could alleviate autoimmune manifestations in MRL/lpr mice. Previous dose–response studies have demonstrated that the route of administration of AhR ligands, extent of AhR activation, and ligand dose might lead to different pharmacokinetics.^14^, ^31^ Our data did not reveal any significant dose‐dependent effects of tapinarof treatment in MRL/lpr mice. The proposed explanations for these differences are the cytokine microenvironment for cell differentiation or the sensitivity of distinct cell types to immune modulation by AhR ligands. Consequently, an optimized dose of tapinarof should be estimated for future clinical use.

The JAK‐STAT signaling pathway plays a critical role in inflammation, immune responses, and autoimmune diseases, including SLE.^32^ Activation of STAT3 is also important for mediating Tfh differentiation by upregulating the expression of BCL6.^33^ In addition, Dang et al.^34^ have confirmed that the activation status of JAK2‐STAT3 signaling could alter the differentiation of Tfh cells. Based on the important role of the JAK2‐STAT3 signaling pathway, we investigated the molecular mechanism of action of tapinarof and demonstrated that tapinarof could inhibit Tfh cells development through the JAK2‐STAT3 signaling pathway to alleviate lupus symptoms in MRL/lpr mice. Thus, future studies should focus on examining the impact of tapinarof co‐treatment with a STAT3 inhibitor on Tfh cell differentiation in a lupus mouse model.

Certain limitations of our study were noted. Flow cytometric stainings should be improved. To discriminate dead and non‐T cells using flow cytometric analysis, a viability dye and T cell specific markers, such as CD3, should be used. Another issue that needs to be addressed relates to the relatively low number of the collected Tfh population that may have influenced the accuracy of the results. Furthermore, the frequency of other Tfh population subsets (CD4+CXCR5+PD‐1+ICOS+) in addition to CD4+CXCR5+PD‐1 and CD4+CXCR5+ICOS+ should be analyzed. Understanding the effects of tapinarof on Tfh cell differentiation during SLE occurrence and development would be of great help toward disease treatment.

## AUTHOR CONTRIBUTIONS

**Ying Zhang**: Writing—review and editing (equal); methodology (lead). **Yanyan Pan**: Methodology (equal); software (equal). **Peiyi Zhang**: Methodology (equal). **Fang Wang**: Methodology (equal); formal analysis (equal). **Ying Han**: Methodology (equal). **Kailin Li**: Methodology (equal); writing—review and editing (equal). **Wen Jiang**: Software (lead). **Jue Wang**: Writing—review and editing (equal). **Yun Luan**: Writing—review and editing (lead). **Qian Xin**: Conceptualization (lead); writing—original draft (lead); formal analysis (lead).

## CONFLICT OF INTEREST STATEMENT

The authors declare no conflict of interest.

## Supporting information

Supporting information.

## Data Availability

The data sets used and analyzed in the manuscript are available from the corresponding author upon reasonable request.
