# Supplementary material for: AhR agonist tapinarof ameliorates lupus autoimmunity by suppressing Tfh cell differentiation via regulation of the JAK2‐STAT3 signaling pathway
Source: Immun Inflamm Dis. 2023 Jun 14;11(6):e903. doi: 10.1002/iid3.903 (PMC10266146; doi:10.1002/iid3.903)
Supplement: Supplementary file 1 — Supporting information. [file IID3-11-e903-s001.pptx]

## Slide 1
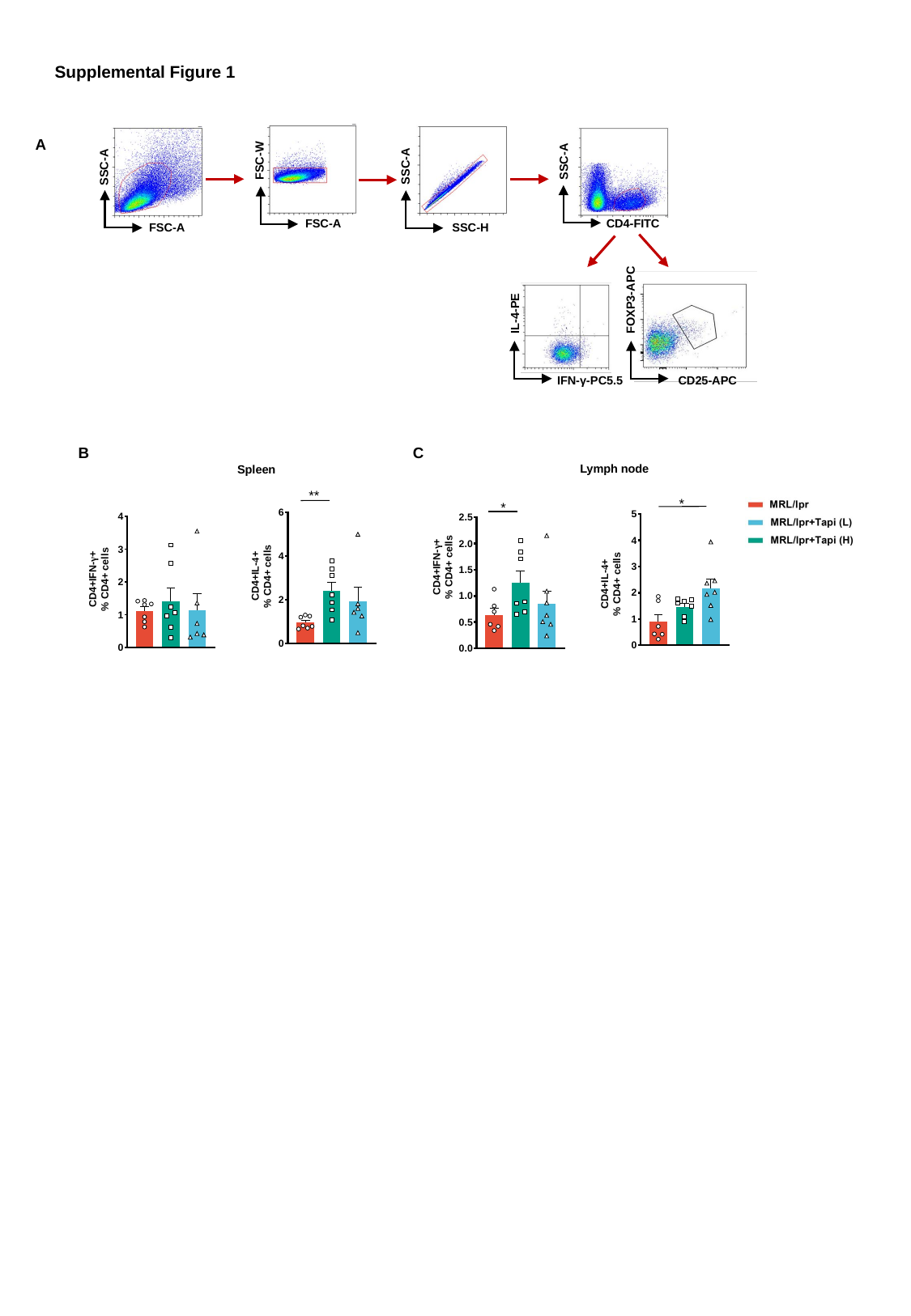

Supplemental Figure 1
A
FSC-W
SSC-A
SSC-A
SSC-A
CD4-FITC
FSC-A
SSC-H
FSC-A
FOXP3-APC
IL-4-PE
IFN-γ-PC5.5
CD25-APC
B
C
Lymph node
Spleen
**
*
*
CD4+IFN-γ+
% CD4+ cells
CD4+IFN-γ+
% CD4+ cells
CD4+IL-4+
% CD4+ cells
CD4+IL-4+
% CD4+ cells

## Slide 2
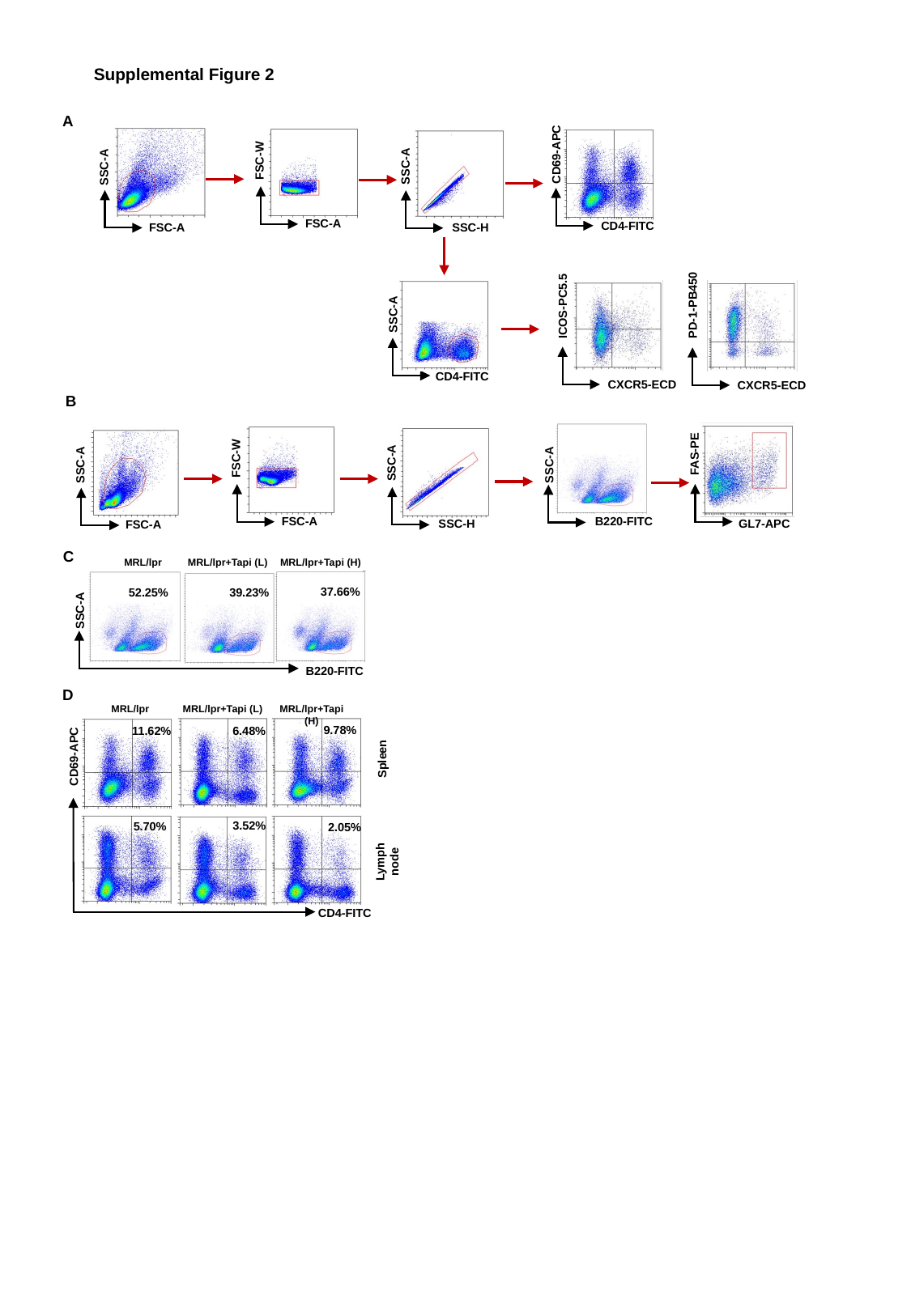

Supplemental Figure 2
A
CD69-APC
FSC-W
SSC-A
SSC-A
FSC-A
CD4-FITC
SSC-H
FSC-A
PD-1-PB450
ICOS-PC5.5
SSC-A
CD4-FITC
CXCR5-ECD
CXCR5-ECD
B
FAS-PE
FSC-W
SSC-A
SSC-A
SSC-A
FSC-A
B220-FITC
SSC-H
GL7-APC
FSC-A
C
MRL/lpr
MRL/lpr+Tapi (L)
MRL/lpr+Tapi (H)
37.66%
39.23%
52.25%
SSC-A
B220-FITC
D
MRL/lpr
MRL/lpr+Tapi (L)
MRL/lpr+Tapi (H)
9.78%
6.48%
11.62%
CD69-APC
Spleen
3.52%
5.70%
2.05%
Lymph node
CD4-FITC

## Slide 3
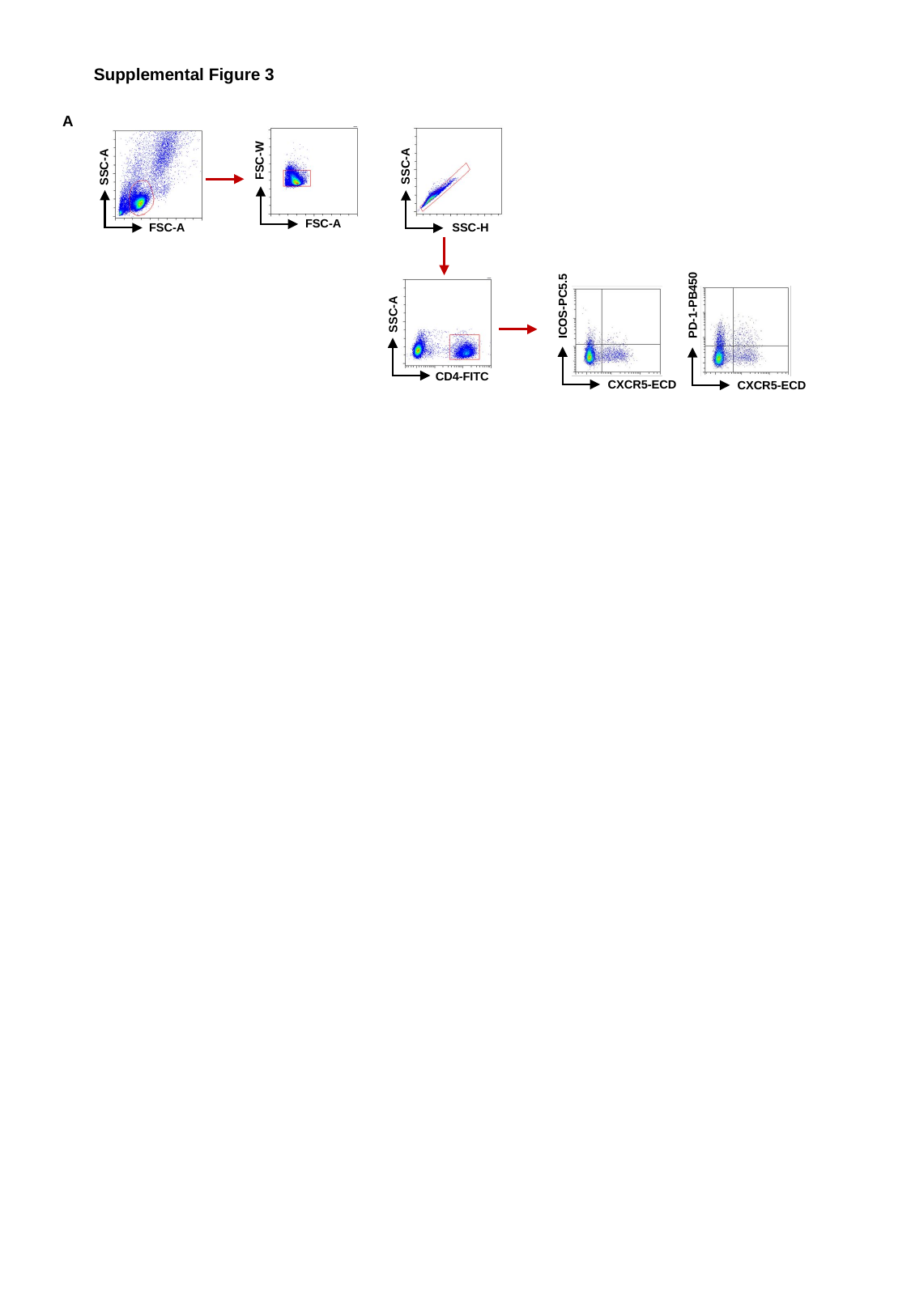

Supplemental Figure 3
A
FSC-W
SSC-A
SSC-A
FSC-A
SSC-H
FSC-A
PD-1-PB450
ICOS-PC5.5
SSC-A
CD4-FITC
CXCR5-ECD
CXCR5-ECD
